# Supplementary figures and images for: The neutrophil-to-lymphocyte ratio as a predictor of all-cause mortality in individuals with anemia: A population-based study
Source: PLoS One. 2026 Jan 16;21(1):e0338129. doi: 10.1371/journal.pone.0338129 (PMC12810842; doi:10.1371/journal.pone.0338129)

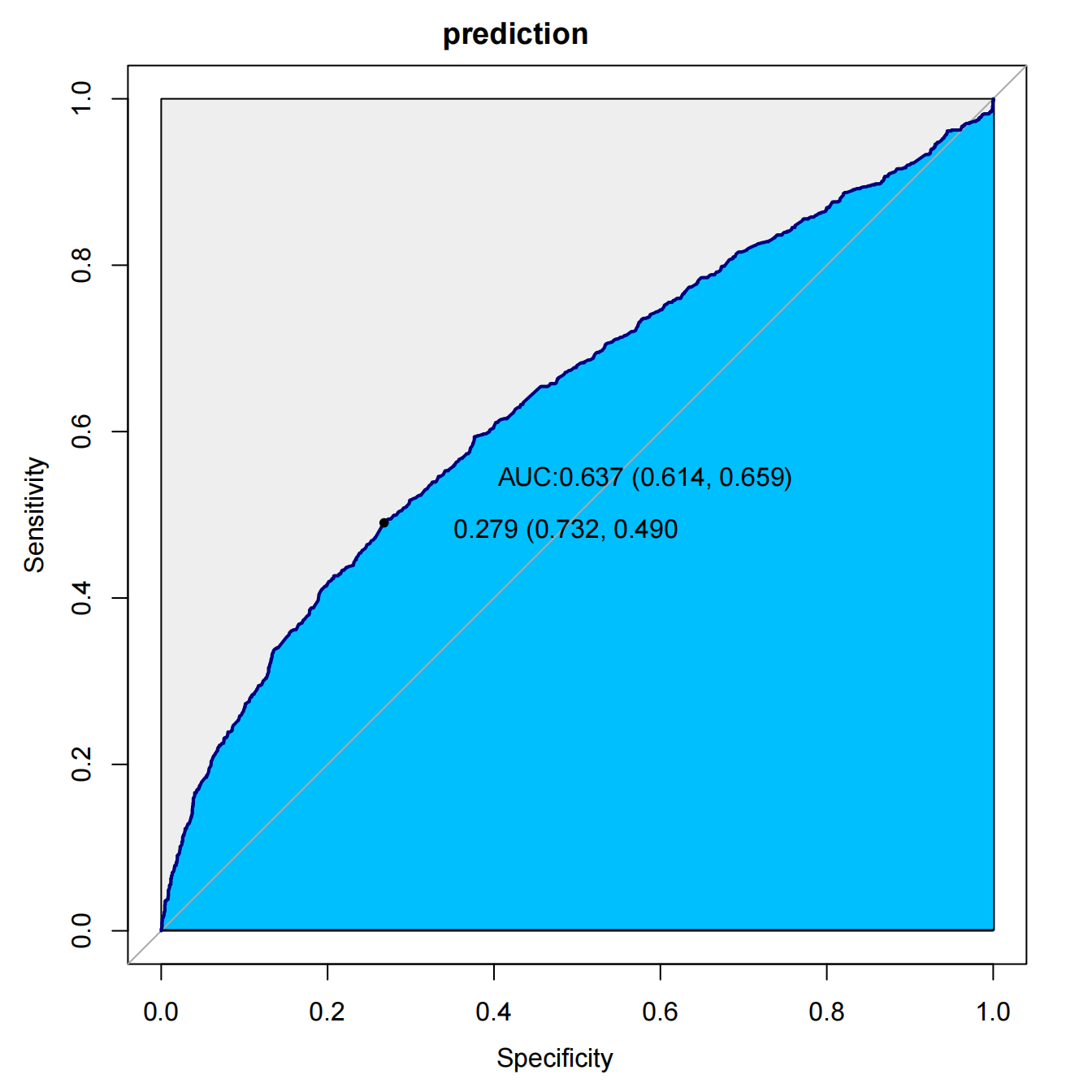

Supplement: S1 Fig — (PNG) [file pone.0338129.s001.png]
