# Supplementary material for: The neutrophil-to-lymphocyte ratio as a predictor of all-cause mortality in individuals with anemia: A population-based study
Source: PLoS One. 2026 Jan 16;21(1):e0338129. doi: 10.1371/journal.pone.0338129 (PMC12810842; doi:10.1371/journal.pone.0338129)
Supplement: Attachment — Submitted filename: Review of Manuscript Number PONE-D-25-19337.docx [file pone.0338129.s002.docx]

**Review of Manuscript Number PONE-D-25-19337**

**"The Neutrophil-to-Lymphocyte Ratio as a Predictor of All-Cause Mortality in Individuals with Anemia: A Population-Based Study"**

Top of Form

Bottom of Form

**Title and Abstract**

Comments:

The title clearly communicates the study design, biomarker, and target population.

Abstract includes all key components: background, methods, results, and conclusion.

However,

Line 50: The term “substantially higher” is vague. Quantify or phrase it more precisely: e.g., “associated with a 25% increase in mortality risk.”

**Introduction**

Comments:

The introduction clearly articulates the clinical burden of anemia and the emerging relevance of NLR.

References are generally up to date.

However,

Line 58: Rather than “simple and widely available,” describe NLR in hematologic terms, e.g., “a leukocyte-derived inflammation index obtainable from a routine complete blood count.”

Line 70: Consider stating the hypothesis explicitly: “We hypothesized that higher NLR would be independently associated with increased all-cause mortality in anemic individuals.”

**Methods**

Comments:

Good explanation of NHANES data source and selection criteria. Clear definition of anemia using WHO guidelines. Use of logistic and Cox models is appropriate.

However,

Line 79: You refer to this as a cross-sectional study. However, the use of follow-up data from NHANES mortality linkage makes this a retrospective cohort analysis. consider revising.

Line 113: NLR Grouping - Tertile cutoffs are not defined. Add the actual NLR ranges for each tertile.

Lines 122-126: Subgroup Analysis - Specify the method used for interaction testing. Was multiplicative interaction tested in the Cox model?

**Results**

Comments:

Tables and figures provide detailed comparisons across NLR tertiles. Clear association between increasing NLR and mortality. Appropriate use of multivariate Cox regression.

However,

Table 1:

Include actual NLR cutoffs for tertiles in the header.

Check formatting: many p-values and spacing issues reduce readability.

Lines 173-182: Report model diagnostics for logistic regression (e.g., AUC, Hosmer-Lemeshow).

Table 2: Include all variable categories in regression models to interpret ORs properly.

Lines 198-204: The non-linear regression results are important, but poorly described. Clarify the method used (e.g., restricted cubic spline) and describe what “breakpoint” means in plain language.

**Discussion**

Comments:

Findings are placed in context with other studies. Clinical relevance of the NLR threshold is emphasized. Limitations are appropriately acknowledged.

However,

Line 225: Opening sentence restates the results; instead, start with interpretation - e.g., “This study shows that inflammation, as measured by NLR, is strongly linked with mortality in individuals with anemia.”

Line 258-264: Discuss whether the NLR threshold of 1.475 has been validated in other studies.

Line 278: “Cross-sectional nature” is incorrect.

Gabriel Abbam (GA)
