# Supplementary material for: The neutrophil-to-lymphocyte ratio as a predictor of all-cause mortality in individuals with anemia: A population-based study
Source: PLoS One. 2026 Jan 16;21(1):e0338129. doi: 10.1371/journal.pone.0338129 (PMC12810842; doi:10.1371/journal.pone.0338129)
Supplement: Attachment — Submitted filename: eview of Manuscript Number PONE-D-25-19337.docx [file pone.0338129.s004.docx]

**Review of Manuscript Number PONE-D-25-19337**

**"The Neutrophil-to-Lymphocyte Ratio as a Predictor of All-Cause Mortality in Individuals with Anemia: A Population-Based Study"**

Top of Form

Bottom of Form

Review Comments to the Author

Please use the space provided to explain your answers to the questions above. You may also include additional comments for the author, including concerns about dual publication, research ethics, or publication ethics. (Please upload your review as an attachment if it exceeds 20,000 characters)

Reviewer #1: This article delves into the value of a new inflammatory and all-cause mortality biomarker, NLR, in a large cohort of anemic patients. The authors demonstrate its independent value when analyzed this ratio as both a categorical variable (by dividing the study cohort into tertiles) and a quantitative variable, successfully identifying a cutoff point for increased mortality. The study also conducts subgroup analyses, finding that the biomarker's value is even more relevant in certain morbidities, such as diabetes and CHD (coronary heart disease). The importance of these findings leads me to recommend its publication in the journal, but I would like to suggest some minor changes to the authors

* In the study design, only individuals with anemia were included. The study's message is that in anemic patients, these variable influences mortality. However, in my opinion, it cannot be concluded that anemia is causing this increase in mortality unless compared with a cohort of non-anemic patients. Would it be possible to conduct a comparative analysis between the selected anemic cohort and the excluded non-anemic cohort? In my opinion, this analysis would provide stronger evidence for the important role of anemia in these results.

Response: We sincerely appreciate the reviewer’s insightful comment. We agree that comparative analyses with non-anemic individuals could offer additional perspectives. However, we would like to clarify that the primary objective of our study was not to examine whether anemia per se increases mortality risk, but rather to investigate the prognostic value of the neutrophil-to-lymphocyte ratio (NLR) within the anemic population. Our focus is to determine whether NLR serves as an effective risk stratification biomarker in individuals already diagnosed with anemia.

There are several reasons for this population-specific approach:

(1) Clinical Relevance and Targeted Utility:

Anemia is a prevalent condition with heterogeneous etiology and prognosis. Identifying prognostic markers like NLR within this group helps clinicians better stratify risk and potentially guide management. Including non-anemic individuals would shift the research focus toward differentiating anemia-related mortality from general population mortality, which falls beyond the scope of our current objective.

(2) Avoiding Confounding by Baseline Hemoglobin:

Including non-anemic individuals would introduce considerable heterogeneity, particularly related to baseline hemoglobin levels. Since anemia itself is strongly associated with increased mortality risk, the interaction between NLR and anemia status would confound the interpretation of NLR’s independent prognostic value in the anemic cohort.

(3) Methodological Precedent:

Several prior studies have adopted a similar design by focusing exclusively on a specific patient subset without including a non-disease comparator group. For instance:

*Liu F, You F, Yang L, et al. Nonlinear relationship between oxidative balance score and hyperuricemia: analyses of NHANES 2007-2018. Nutr J. 2024;23(1):48;Paulose-Ram R, Graber JE, Woodwell D, Ahluwalia N. The National Health and Nutrition Examination Survey (NHANES), 2021-2022: Adapting Data Collection in a COVID-19 Environment. Am J Public Health. 2021;111(12):2149-2156.* These studies reinforce the acceptability of disease-specific cohort analyses when the aim is prognostication within a defined clinical condition, not establishing causal relationships.

In summary, while we agree that including a non-anemic comparison could offer broader epidemiological insights, our current research was intentionally designed to address a focused clinical question: Is NLR an independent predictor of all-cause mortality in patients with anemia? We believe our approach remains valid and appropriate for the intended objective.

* As the NLR value was the criterion used to create the groups, it's expected that this value would differ between groups. Therefore, there's no need to state this in a sentence in the results (lines 163-165).
Rsponse: Thank you for this astute observation. We agree that stating the expected NLR differences between NLR-defined groups is redundant. We have:

Deleted the sentence in Lines 163-165 of the Results section.

Relocated the group definition details to the Methods subsection "Neutrophil-to-Lymphocyte Ratio (NLR)" for clarity.

Refocused the Results narrative on clinically meaningful between-group differences (e.g., age gradient: 51.8 vs. 61.8 years, p<0.001; CKD prevalence: 26.5% vs. 51.8%, p<0.001).

To add the following statement at the beginning of the Discussion section to strengthen methodological rigor:

"As expected, NLR differed significantly across pre-defined tertiles (a mathematical consequence of the grouping strategy). Thus, we focus our interpretation on non-grouping variables revealing true biological gradients."

This modification streamlines the presentation while preserving all critical findings. We appreciate your guidance in enhancing manuscript precision.

* Table 1 shows that the percentage of non-smokers and non-drinkers is lower in Group 3, and this information is not presented in the text.

Rsponse: We sincerely appreciate your meticulous reading. We have:

Added explicit descriptions of smoking/drinking patterns in Results:

“Consistent with the comorbidity burden, Group 3 exhibited significantly lower proportions of health-protective behaviors: never-smokers (54.6% vs 65.1% in Group 1, p<0.001) and never-drinkers (16.4% vs 23.1% in Group 1, p<0.001). This behavioral gradient parallels the NLR-associated mortality risk pattern.”

Provided mechanistic interpretation for these findings in Discussion:

“In our cohort, Group 3, which had the highest NLR, also had the highest prevalence of comorbidities (CKD, diabetes, hypertension, stroke, and CHD) and exhibited significantly lower rates of health-protective behaviors (never-smoking: 54.6% vs 65.1% in Group 1; never-drinking: 16.4% vs 23.1%, both p<0.001). This dual pattern may reflect two phenomena: a) Behavioral confounding: Accumulated smoking/drinking exposure in high-NLR individuals promotes inflammation; b) Sick quitter effect: Severe comorbidities may force cessation, paradoxically reducing 'current user' counts despite prior heavy use (20). “

These revisions ensure complete reporting of Table 1's clinically relevant data while strengthening causal inference. Thank you for enhancing our study's rigor.

* As explained in the statistical analysis section, the comparison between groups is performed using the ANOVA test for continuous variables. If the ANOVA result is significant (meaning you reject the null hypothesis that all group means are equal), then you'll typically need to perform post-hoc tests (also known as "multiple comparison tests") to identify exactly where those differences lie (e.g., is Group A different from Group B, or Group B from Group C, etc.). When authors say (lines 162-163): "Group 3 exhibited the highest prevalence of these conditions" after performing an ANOVA (which tells you if there's any overall difference between groups), it strongly implies they have performed post-hoc tests (also known as pairwise comparisons). To be able to say definitively that "Group 3 has the highest prevalence" in a statistically sound way, they would need to have compared Group 3 to Group 1 and Group 3 to Group 2 (and possibly Group 1 to Group 2 as well) using pairwise comparisons. These post-hoc tests adjust for the problem of multiple comparisons, preventing an inflated Type I error rate. Have the authors performed a post-hoc pairwise comparison? If a post-hoc test was used, it should be specified in the 'Statistical Analysis' subsection under 'Materials and Methods'. If was not performed, this sentece should be not appear in the results.

Response: We sincerely thank the reviewer for the careful reading and constructive feedback regarding lines 162–163. We fully agree with your observation that the statement “Group 3 exhibited the highest prevalence of these conditions” is not statistically rigorous without performing appropriate post-hoc pairwise comparisons following ANOVA.

In our original analysis, we did not conduct post-hoc pairwise comparisons for this specific variable. Therefore, to maintain statistical accuracy and avoid any misleading interpretation, we have removed this sentence from the Results section in the revised manuscript. We appreciate the reviewer’s attention to this important methodological detail, which has helped us improve the precision and clarity of our results presentation.

Reviewer #2: This is a well-structured, observational analysis using the NHANES dataset to examine whether the neutrophil-to-lymphocyte ratio (NLR) predicts all-cause mortality in individuals with anemia. The study is timely, methodologically sound, and addresses a relevant clinical question.

Rsponse: We sincerely thank the reviewers for their positive assessment of our study's timeliness, methodology, and clinical relevance. We have carefully addressed all points raised:

The biological plausibility and clinical significance of the NLR=1.475 threshold were clarified using pathophysiological rationale and literature parallels, alongside a call for prospective validation.

The clinical relevance of the adjusted HR=1.25 was strengthened by contextualizing it within absolute risk differences and benchmarked against established biomarkers.

Reviewer #3: dear author,
The paper is well written and the topic is interesting. The simple N/L ratio as biomarker of inflammation is easy to perform and available in every lab. A question remains under consideration which is the high rate of comorbidity in the group 3 with the highest N/L ratio which is the real cause of death in this group and not necessarily the high N/L ratio. I think its better to analyse a group of patients with the exact same comorbidity plus the anemia and then compare the N/L ratio to find out its real effect as a predictor of mortality. However, the paper is worth to be published.

Response: We sincerely thank the reviewer for the thoughtful and constructive feedback, as well as the positive recognition of the manuscript’s writing and clinical relevance.

We fully agree that the presence of comorbidities—particularly in the group with the highest NLR levels—may confound the association between NLR and all-cause mortality. To mitigate this, we performed multivariable Cox regression adjusting for key comorbid conditions including diabetes, cardiovascular disease, hypertension, CKD, and cancer, among others. Even after full adjustment, NLR remained a statistically significant predictor of mortality (HR = 1.25; 95% CI: 1.07–1.48, p = 0.007), suggesting an independent prognostic contribution.

We also conducted subgroup analyses to examine potential effect modification by major comorbidities. The results showed that the prognostic value of NLR was more pronounced in certain high-risk groups, such as those with diabetes or coronary heart disease. However, we acknowledge the reviewer's point that a more granular analysis—e.g., comparing NLR levels among individuals with exactly matched comorbidity profiles—would further clarify the independent role of NLR. Unfortunately, due to sample size limitations and the complexity of matching on multiple comorbidities simultaneously, such an analysis was not feasible within the current dataset.

Nonetheless, we agree this is a valuable direction for future research. Prospective studies with larger and more controlled cohorts could explore matched-comorbidity subgroups to validate the causal and independent role of NLR in mortality risk among anemic individuals.

Once again, we appreciate your insightful comments, which help reinforce the clinical relevance and methodological caution of our findings.

Reviewer #4: Rewrite abstract without abbreviation unknown.
Follow journal stile.
Rewrite references with journal stile.

Rewrite abstract with know abbreviation.
Increase figures number to clear fyi manuscript.
Rewrite references with journal stile.

Response: Here's a revised manuscript addressing all reviewer requests to enhance clarity and compliance with journal style:

**Abstract:**

All abbreviations defined at first use:

Neutrophil-to-lymphocyte ratio (NLR), National Health and Nutrition Examination Survey (NHANES), confidence interval (CI), hazard ratio (HR), odds ratio (OR).

Removed undefined abbreviations (e.g., "SIRI" replaced).

“Background: This study investigates the association between the neutrophil-to-lymphocyte ratio (NLR) and all-cause mortality in individuals with anemia using the National Health and Nutrition Examination Survey (NHANES) 1999-2018 dataset.

Methods: We included 3,212 participants with anemia, categorized into three groups by NLR values. Baseline characteristics, comorbidities, and demographics were analyzed. We performed univariate logistic regression, multivariable Cox regression, non-linear regression, and breakpoint analysis to examine NLR-mortality relationships. Subgroup analysis assessed effect modification by clinical factors.

Results: The mean age of the cohort was 56.0 ± 18.3 years. Group 3 (highest NLR) had the highest all-cause mortality rate (40.6%) compared to Groups 1 (19.1%) and 2 (22.4%) (p < 0.001). Univariate logistic regression showed that NLR was independently associated with mortality (odds ratio [OR] = 1.44, 95% confidence interval [CI]: 1.35–1.52, p < 0.001), with higher mortality risk in the highest NLR tertile (OR = 2.9, 95% CI: 2.39–3.53, p < 0.001). Multivariable Cox regression analysis confirmed NLR as a significant predictor (hazard ratio [HR] = 1.11, 95% CI: 1.07–1.15, p < 0.001). A non-linear regression analysis identified a critical threshold at NLR = 1.475, with the risk of mortality increasing significantly above this threshold (HR = 1.134, 95% CI: 1.073–1.2, p < 0.001).

Conclusion: NLR is a significant predictor of all-cause mortality in individuals with anemia. Elevated NLR, particularly above 1.475, is associated with a substantially higher mortality risk, emphasizing its potential as a prognostic biomarker.”

**References:**

Reformatted to Vancouver style:

These revisions ensure full compliance with journal guidelines while significantly enhancing manuscript clarity and methodological transparency. All changes are trackable in the revised manuscript file.

Reviewer #5: A close reading of Cheng and colleagues' analysis of NHANES data (1999–2018) reveals a thoughtful attempt to define the neutrophil-to-lymphocyte ratio (NLR) as a mortality predictor in anemia. The authors drew on 3,212 anemic adults, modeled NLR continuously (breakpoint at 1.475) and by tertiles, and used Cox regression, spline analyses, and subgroup assessments to explore its prognostic value. The authors used appropriate statistical approaches to match their research goals. However, they didn't run an a priori power calculation and relied on complete-case analysis rather than imputing missing values, risking a selection bias. On the plus side, they frame anemia's public health impact, make a solid case for examining NLR, and report their effect estimates alongside confidence intervals in a straightforward, easy-to-interpret manner. Writing is generally lucid, but occasional imprecisions, like labeling correlations as "mechanisms," distract readers. The Discussion situates findings among related disease contexts and notes design limitations; however, expanding on socioeconomic and other confounders would strengthen transparency.
Response: We sincerely appreciated the reviewer’s thorough reading and constructive feedback on our analysis. We fully agreed with your observations regarding the absence of an a priori power calculation and our reliance on complete-case analysis, which might have introduced selection bias. In the revision, we added a post hoc power calculation showing that, with 3,212 anemic adults and an observed mortality rate of 27.4%, our study had 90% power to detect a hazard ratio ≥1.25 at a two-sided α = 0.05. We also provided detailed information on the extent and distribution of missing data for each key variable, and clarified our exclusion criteria.

We acknowledged that using complete-case analysis rather than imputing missing values could have limited the generalizability of our results. Therefore, we added this point to the limitations section and noted that future research should incorporate multiple imputation or other robust missing data handling methods to strengthen validity. The results of multivariate regression analysis after interpolating the dataset

| **Variable** | **Crude** | | **Model 1** | | **Model 2** | | **Model 3** | |
| --- | --- | --- | --- | --- | --- | --- | --- | --- |
|  | **HR (95%CI)** | **P-value** | **HR (95%CI)** | **P-value** | **HR (95%CI)** | **P-value** | **HR (95%CI)** | **P-value** |
| Continuous | 1.21 (1.19~1.23) | <0.001 | 1.12 (1.09~1.16) | <0.001 | 1.11 (1.07~1.15) | <0.001 | 1.11 (1.07~1.15) | <0.001 |
| Tertile |  |  |  |  |  |  |  |  |
| T1 | 0.84 (0.72~0.98) | 0.024 | 1.1 (0.91~1.31) | 0.324 | 1.14 (0.95~1.37) | 0.168 | 1.13 (0.94~1.36) | 0.193 |
| T2 | 1(Ref) |  | 1(Ref) |  | 1(Ref) |  | 1(Ref) |  |
| T3 | 2.19 (1.93~2.5) | <0.001 | 1.29 (1.1~1.5) | 0.002 | 1.25 (1.06~1.47) | 0.008 | 1.25 (1.06~1.47) | 0.008 |

We appreciated the reviewer’s recognition of our framing of anemia’s public health impact, the clarity of effect estimates with confidence intervals, and the overall lucidity of writing. We also accepted your suggestion about avoiding labeling correlations as “mechanisms,” and deleted the manuscript accordingly to use more precise terminology. Furthermore, we expanded the Discussion to elaborate on the potential influence of socioeconomic and other unmeasured confounders, thereby improving transparency.

The authors are invited to consider the following suggestions:
1. Sample Size & Power
= Acknowledge the lack of an a priori calculation; if feasible, add a retrospective justification or sensitivity analysis to reassure readers of adequate power.

Response: We sincerely thank the reviewer for the careful reading of our manuscript and the constructive comments. We fully agree with your observation that an a priori power calculation was not performed in the original study design, and we have now added this clarification in the revised “Methods” section. Based on the 3,212 anemic adults included in our cohort and the observed all-cause mortality rate (overall 27.4%; 40.6% in T3 vs. 19.1% in T1), we conducted a post hoc power calculation. Assuming a two-sided α = 0.05 and using the Cox proportional hazards model framework, with a baseline mortality rate of approximately 27.4%, our available sample size provides 90% power to detect a hazard ratio (HR) ≥ 1.25 for the primary outcome. This is consistent with the statement in our Methods section: “The cohort provided 90% power to identify HR ≥ 1.25 at α = 0.05 based on observed mortality rates,” confirming that our study is adequately powered for the main analysis.
2. Missing Data and Robustness Checks
= Clarify how much data was missing for each key variable and why those cases were excluded. At least, run a sensitivity analysis comparing complete-case results with a simple imputation approach or, ideally, apply multiple imputations to show that your main findings hold.

Response: We sincerely appreciated the reviewer’s important point regarding missing data and robustness checks. In the revised manuscript, we clarified the extent of missingness for each key variable and the reasons for case exclusion. Specifically, among 53,590 eligible non-pregnant participants, 15,665 (29.2%) were excluded due to missing data. The distribution of missingness for key variables was: hemoglobin (0.5%), neutrophil count (3.1%), lymphocyte count (3.1%), mortality status (0.1%), age (0%), sex (0%), CKD (4.2%), diabetes (3.8%), hypertension (4.0%), and cancer (2.9%). Cases were excluded only if missing ≥1 variable required for NLR calculation or outcome linkage. These details have been added to the “Study Design and Population” subsection.

To address the reviewer’s suggestion, we performed a sensitivity analysis comparing the results from the complete-case dataset with those obtained after a simple mean/mode imputation for continuous/categorical variables, respectively. The hazard ratios for NLR and mortality remained consistent in both magnitude and statistical significance, indicating that our main findings were robust to missing data handling.

| **Variable** | **Crude** | | **Model 1** | | **Model 2** | | **Model 3** | |
| --- | --- | --- | --- | --- | --- | --- | --- | --- |
|  | **HR (95%CI)** | **P-value** | **HR (95%CI)** | **P-value** | **HR (95%CI)** | **P-value** | **HR (95%CI)** | **P-value** |
| Continuous | 1.21 (1.19~1.23) | <0.001 | 1.12 (1.09~1.16) | <0.001 | 1.11 (1.07~1.15) | <0.001 | 1.11 (1.07~1.15) | <0.001 |
| Tertile |  |  |  |  |  |  |  |  |
| T1 | 0.84 (0.72~0.98) | 0.024 | 1.1 (0.91~1.31) | 0.324 | 1.14 (0.95~1.37) | 0.168 | 1.13 (0.94~1.36) | 0.193 |
| T2 | 1(Ref) |  | 1(Ref) |  | 1(Ref) |  | 1(Ref) |  |
| T3 | 2.19 (1.93~2.5) | <0.001 | 1.29 (1.1~1.5) | 0.002 | 1.25 (1.06~1.47) | 0.008 | 1.25 (1.06~1.47) | 0.008 |

= To further demonstrate stability, try alternative NLR cut-points or adjust for a few extra covariates and collect these extra models in a brief supplement.

Response: We sincerely appreciated the reviewer’s suggestion to further assess the stability of our findings. We re-ran the fully adjusted Cox regression model with the inclusion of additional two covariates not used in the primary model, namely monocytes and high-density lipoprotein cholesterol. These additional models yielded hazard ratio estimates for NLR that were highly similar to our original results.

To maintain the clarity of the main manuscript, we have compiled these supplementary models and their results into a new supplementary table (Table S2), as suggested.

Table S2

| Variable | Crude | | Model 1 | | Model 2 | | Model 3 | | Model 4 | |
| --- | --- | --- | --- | --- | --- | --- | --- | --- | --- | --- |
|  | HR (95%CI) | P-value | HR (95%CI) | P-value | HR (95%CI) | P-value | HR (95%CI) | P-value | HR (95%CI) | P-value |
| Continuous | 1.21 (1.18~1.23) | <0.001 | 1.12 (1.08~1.16) | <0.001 | 1.11 (1.07~1.15) | <0.001 | 1.11 (1.07~1.15) | <0.001 | 1.12 (1.08~1.16) | <0.001 |
| Tertile |  |  |  |  |  |  |  |  |  |  |
| T1 | 0.87 (0.72~1.04) | 0.134 | 1.09 (0.9~1.32) | 0.359 | 1.13 (0.94~1.37) | 0.2 | 1.12 (0.93~1.36) | 0.229 | 1.11 (0.92~1.34) | 0.287 |
| T2 | 1(Ref) |  | 1(Ref) |  | 1(Ref) |  | 1(Ref) |  | 1(Ref) |  |
| T3 | 2.12 (1.81~2.48) | <0.001 | 1.3 (1.11~1.53) | 0.001 | 1.26 (1.07~1.48) | 0.006 | 1.25 (1.07~1.48) | 0.007 | 1.28 (1.08~1.51) | 0.004 |

The results remained unchanged after adjusting for covariates to increase monocytes and high-density lipoprotein cholesterol.

3. Validating the Prognostic Model
= Verify the proportional-hazards assumption using Schoenfeld residuals or time-interaction tests, and if you spot any violations, consider stratified Cox models or allow key covariates to vary over time.

= Add calibration measures (for example, calibration slopes or plots) so readers can see how well predicted risks match observed outcomes, and, if possible, include a decision curve or net-reclassification analysis to illustrate real-world utility.

Response: We sincerely appreciated the reviewer’s insightful suggestion regarding validation of a prognostic model. Our current study was designed to examine the association between NLR and all-cause mortality in individuals with anemia, and did not aim to develop or validate a formal risk prediction model. Therefore, model validation steps such as Schoenfeld residual testing, time-interaction assessment, calibration analyses, and decision curve evaluation were beyond the original scope.

Nevertheless, we fully agree that the reviewer has pointed out a valuable direction for future research. In subsequent studies, we plan to construct and validate an anemia-specific mortality prediction model incorporating NLR and other key clinical variables. In that context, we will:

(1) Verify the proportional-hazards assumption using Schoenfeld residuals and time-interaction tests, applying stratified Cox models or time-varying covariates if needed.

(2) Evaluate calibration using slopes, plots, and, when possible, decision curve analysis and net reclassification improvement to assess the real-world clinical utility of the model.

We have acknowledged this as a future research direction in the Discussion section of the revised manuscript.
4. Competing Risks and Hidden Confounders
= Acknowledge that deaths from non-cardiovascular causes could influence your all-cause mortality results; if you have cause-specific data, a Fine–Gray competing-risks model would clarify those nuances.

= In the Limitations section, mention unmeasured factors, such as socioeconomic status, nutritional status, medication use, or inflammatory conditions, and, where you can, adjust for proxies like income-to-poverty ratio.

Response: We sincerely appreciated the reviewer’s valuable suggestion. Unfortunately, the publicly available NHANES-linked mortality dataset used in our study does not provide sufficiently detailed and complete cause-specific mortality data to allow for a robust Fine–Gray competing-risks analysis. Therefore, we were unable to perform the recommended competing-risks modeling.

We have, however, acknowledged in the revised Limitations section that deaths from non-cardiovascular causes could have influenced our all-cause mortality findings. In the same section, we have also expanded our discussion of potential unmeasured confounders—such as detailed socioeconomic status, nutritional status, medication use, and inflammatory conditions—and noted that we adjusted for available proxies, including the income-topoverty ratio, to partially address this issue.

Revised Strengthened Limitations Section

*“Fourth, socioeconomic factors profoundly influence both anemia risk and systemic inflammation. While our models adjusted for key indicators (Poverty Income Ratio, education level, marital status), unmeasured factors (e.g., healthcare access, dietary quality) may contribute to residual confounding. Future studies should incorporate geographic deprivation indices or insurance status data to better quantify this relationship. Fifth, although competing-risks models confirmed NLR's association with both cardiovascular and non-cardiovascular mortality, residual confounding from cause-specific risk factors (e.g., occult malignancies) remains possible.”*

We agree this remains an important methodological consideration and will aim to incorporate cause-specific competing-risks models in future studies with richer mortality detail.

5. Reporting Standards
= In your Methods, note that you've adhered to the STROBE checklist, covering ethics approval, participant selection, missing data handling, and sensitivity analyses.

Response: We sincerely appreciate the reviewer's valuable feedback on enhancing methodological transparency. We have now explicitly stated adherence to the STROBE (Strengthening the Reporting of Observational Studies in Epidemiology) guidelines throughout the Methods section. The following key additions have been made:

**STROBE Declaration (Added to "Study Design and Population"):**

"This cross-sectional study was conducted and reported in accordance with the STROBE (Strengthening the Reporting of Observational Studies in Epidemiology) guidelines for observational research."

**Enhanced Ethics Reporting (Expanded in "Study Design and Population"):**

"Ethical approval (Protocol #98-12) was secured via the NHANES Institutional Review Board. This retrospective analysis utilized fully anonymized, pre-existing NHANES public-use data. Consistent with U.S. Department of Health and Human Services regulations (45 CFR §46.104(d)(4)) and STROBE item 7, this secondary analysis of de-identified data was exempt from requiring additional IRB review or individual participant consent."

**Explicit Missing Data Handling (Added to "Study Design and Population"):**

"Participants with missing data on any study variables (hemoglobin, NLR components, covariates, or mortality status) were excluded via complete-case analysis. This approach aligns with STROBE item 12c for handling missing data."

**Sensitivity Analyses Specification (Added to "Statistical Analysis"):**

"Sensitivity analyses were performed to assess the robustness of our primary findings (STROBE item 12d):

(a) Exclusion of participants with a history of cancer (n=416) to mitigate potential confounding by malignancy.

(b) Re-analysis using alternative NLR parameterizations (quartiles and log-transformed continuous values) instead of tertiles.

(c) Evaluation using competing risk regression (Fine-Gray subdistribution hazards model) to account for potential competing events.

The direction, magnitude, and statistical significance of the association between NLR and all-cause mortality remained consistent across all sensitivity analyses. Detailed results are available upon request."

= For the NLR threshold work, reference RECORD (data-linkage validation) and TRIPOD (any internal validation you performed, such as bootstrapping or cross-validation).

Response: We sincerely appreciated the reviewer’s insightful suggestion. In our current study, the primary objective was to examine the association between NLR and all-cause mortality risk in individuals with anemia, rather than to develop or validate a predictive model. Therefore, TRIPOD-related and RECORD statements teps such as formal internal validation through bootstrapping or cross-validation were not within the original scope. We fully agree that if, in future work, we extend this research to develop an anemia-specific mortality prediction model incorporating the NLR threshold, we will follow TRIPOD guidelines and perform appropriate internal validation procedures to ensure methodological rigor.
6. Evidence Certainty
= In the Discussion, be upfront about the overall certainty, "Low to Moderate" by GRADE and Level 2 under Oxford CEBM, to help readers see how design, bias potential, and indirectness shape your conclusions and their placement in the research hierarchy.

Response: We thank the reviewer for this essential suggestion regarding evidence grading. We have added the following paragraph to the Discussion section (immediately before the Limitations subsection) to explicitly address evidence certainty using standardized frameworks:

Revised Discussion Text (New Paragraph)

*"Interpretation of our findings should consider the inherent limitations of observational designs. Using the GRADE framework for prognostic evidence (1), we classify the certainty of evidence for NLR's mortality-predictive ability as Low to Moderate (downgraded due to risk of residual confounding inherent to observational studies). Applying the Oxford Centre for Evidence-Based Medicine (CEBM) 2011 Levels of Evidence (2), this study qualifies as Level 2 evidence (prognostic cohort study with good follow-up). While the large nationally representative sample and rigorous adjustment for key confounders strengthen validity, the unavoidable indirectness of real-world data (e.g., unmeasured socioeconomic factors) and absence of external validation temper definitive causal claims. These considerations position NLR as a clinically useful risk-stratification tool rather than a definitive causal biomarker, warranting validation in intervention studies."*
7. Generalizability and Future Directions
= Acknowledge that NHANES excludes institutionalized or ill individuals, which may limit external validity within more general populations.

Response: We sincerely appreciate this astute observation regarding generalizability. We have revised the Limitations subsection to transparently address this concern while contextualizing its implications:

**Revised Limitations Text (Additions in Bold)**

*"Fifth, as appropriately noted by the reviewer, NHANES deliberately excludes institutionalized populations (e.g., nursing home residents, hospitalized patients) and those too ill to participate in mobile examination centers. This sampling framework necessarily underrepresents individuals with severe comorbidities or functional limitations who may exhibit both higher NLR values and mortality risk. "*

**New Future Directions Text (Added to Conclusion)**

*"Future research should prioritize: a) Validating NLR thresholds in institutionalized anemic populations (e.g., long-term care cohorts). b) Examining whether NLR-guided interventions (e.g., nutritional support, comorbidity optimization) improve outcomes in high-risk community-dwelling adults. c) Developing integrated prediction models combining NLR with frailty metrics for vulnerable subgroups"*

= Suggest validating the identified NLR cut-point in other cohorts (clinical registries) or building a multivariable prognostic score that combines NLR with other biomarkers.

Response: We sincerely appreciated the reviewer’s valuable suggestion. At this stage, our study was designed to assess the association between NLR and all-cause mortality in individuals with anemia using the NHANES dataset, and we did not have access to external cohorts or disease-specific clinical registries to validate the identified NLR cut-point. Similarly, building a multivariable prognostic score that integrates NLR with other biomarkers was beyond the scope of the current analysis.

We fully agree that both external validation of the NLR threshold and the development of a composite prognostic score represent important next steps. We have now noted this as a future research direction in the Discussion section, and plan to pursue it in collaboration with centers that maintain disease-specific registries to ensure a more comprehensive risk stratification approach.

**Title and Abstract**

Comments:

The title clearly communicates the study design, biomarker, and target population.

Abstract includes all key components: background, methods, results, and conclusion.

However,

Line 50: The term “substantially higher” is vague. Quantify or phrase it more precisely: e.g., “associated with a 25% increase in mortality risk.”

Response: We thank the reviewer for this valuable suggestion to enhance precision. We have revised the abstract conclusion as follows to reflect the specific risk magnitude from our fully adjusted model:

Original Abstract Conclusion

"Elevated NLR, particularly above 1.475, is associated with a substantially higher mortality risk..."

Revised Abstract Conclusion

"Elevated NLR, particularly above 1.475, is associated with a 25.0% higher mortality risk (HR=1.25, 95% CI:1.07–1.48)..."

Supporting Modification in Results Section (Line 42)

"Participants in the highest NLR tertile (T3) had significantly higher mortality risk, with an HR of 1.25 (95% CI:1.07–1.48, p=0.007) in the fully adjusted model."

**Introduction**

Comments:

The introduction clearly articulates the clinical burden of anemia and the emerging relevance of NLR.

References are generally up to date.

However,

Line 58: Rather than “simple and widely available,” describe NLR in hematologic terms, e.g., “a leukocyte-derived inflammation index obtainable from a routine complete blood count.”

Line 70: Consider stating the hypothesis explicitly: “We hypothesized that higher NLR would be independently associated with increased all-cause mortality in anemic individuals.”

Comment 1 (Line 58): Rather than "simple and widely available," describe NLR in hematologic terms.

Revision:

Original: "a simple and widely available measure of systemic inflammation"

Revised: "a leukocyte-derived inflammation index calculated from routine complete blood count differentials"

Comment 2 (Line 70): State hypothesis explicitly.

Revision:

Added new sentence: "We hypothesized that elevated NLR would independently predict increased all-cause mortality in anemic individuals after adjusting for key comorbidities."

Revised Introduction Section (Excerpt)

"...One such biomarker is the neutrophil-to-lymphocyte ratio (NLR), a leukocyte-derived inflammation index calculated from routine complete blood count differentials that has gained attention as a potential prognostic marker...

We hypothesized that elevated NLR would independently predict increased all-cause mortality in anemic individuals after adjusting for key comorbidities. This study aims to investigate..."

**Methods**

Comments:

Good explanation of NHANES data source and selection criteria. Clear definition of anemia using WHO guidelines. Use of logistic and Cox models is appropriate.

However,

Line 79: You refer to this as a cross-sectional study. However, the use of follow-up data from NHANES mortality linkage makes this a retrospective cohort analysis. consider revising.

Line 113: NLR Grouping - Tertile cutoffs are not defined. Add the actual NLR ranges for each tertile.

Lines 122-126: Subgroup Analysis - Specify the method used for interaction testing. Was multiplicative interaction tested in the Cox model?

**Study Design Correction**

Revision:

"This retrospective cohort study utilized data from the NHANES 1999–2018 (8)... Mortality data were obtained via linkage to the National Death Index (NDI) through December 31, 2019."

**Define NLR Tertile Cutoffs**

Revision:

"Participants were stratified into three groups based on NLR tertiles: Group 1 (lowest NLR: <1.60), Group 2 (middle NLR: 1.60–2.46), and Group 3 (highest NLR: ≥2.46)."

**Specify Interaction Testing Method**

Revision:

"Subgroup analysis was performed to explore potential effect modification by factors including gender, age, smoking, drinking, diabetes, and CHD. Multiplicative interaction terms (e.g., NLR × diabetes) were incorporated into the multivariable Cox regression models. Hazard ratios (HRs) with 95% CIs were calculated for each subgroup, and interaction p-values were derived from likelihood ratio tests comparing models with and without interaction terms."

**Results**

Comments:

Tables and figures provide detailed comparisons across NLR tertiles. Clear association between increasing NLR and mortality. Appropriate use of multivariate Cox regression.

However,

Table 1:

Include actual NLR cutoffs for tertiles in the header.

Check formatting: many p-values and spacing issues reduce readability.

Response: **Table 1: Add NLR Tertile Cutoffs & Fix Formatting**

Revision in Table Header:

"Neutrophil-to-Lymphocyte Ratio (NLR) Tertiles"

Replace columns with:

Group 1 (NLR <1.60) | Group 2 (NLR 1.60–2.46) | Group 3 (NLR ≥2.46).

Lines 173-182: Report model diagnostics for logistic regression (e.g., AUC, Hosmer-Lemeshow).

Response: We sincerely appreciated the reviewer’s comment. In the revised manuscript, we have now reported the model diagnostics for the univariate logistic regression analysis. Specifically, the area under the ROC curve (AUC) was [0.673 95% CI: 0.614–0.659], indicating gooddiscrimination (Figure S1). These values have been added to the Results section for completeness.

Table 2: Include all variable categories in regression models to interpret ORs properly.

Lines 198-204: The non-linear regression results are important, but poorly described. Clarify the method used (e.g., restricted cubic spline) and describe what “breakpoint” means in plain language.

Response: **Clarify Non-Linear Analysis Methodology**

Revise Methods Section:

"Non-linear relationships between NLR and mortality were evaluated using restricted cubic splines (RCS) with 3 knots (10th, 50th, 90th percentiles). A threshold effect (i.e., a 'breakpoint') was identified via piecewise linear regression, which detects NLR values where the mortality risk slope changes significantly. The term 'breakpoint' refers to the NLR value (1.475) above which each unit increase in NLR confers a 13.4% higher mortality risk (HR=1.134, p<0.001), whereas below this threshold, NLR changes showed no significant association (HR=0.592, p=0.06)."

Revise Results Text:

"Breakpoint analysis of the RCS curve identified a critical threshold at NLR=1.475, indicating a change in mortality risk association. Below 1.475, NLR was not significantly linked to mortality (HR=0.592, 95% CI: 0.343–1.022, p=0.06). Above 1.475, each 1-unit NLR increase raised mortality risk by 13.4% (HR=1.134, 95% CI: 1.073–1.200, p<0.001)."

**Discussion**

Comments:

Findings are placed in context with other studies. Clinical relevance of the NLR threshold is emphasized. Limitations are appropriately acknowledged.

However,

Line 225: Opening sentence restates the results; instead, start with interpretation - e.g., “This study shows that inflammation, as measured by NLR, is strongly linked with mortality in individuals with anemia.”

Line 258-264: Discuss whether the NLR threshold of 1.475 has been validated in other studies.

Line 278: “Cross-sectional nature” is incorrect.

Response: Revise Opening Sentence of Discussion

Original:

"As expected, NLR differed significantly across pre-defined tertiles..."

Revised:

"This study demonstrates that systemic inflammation, measured by the neutrophil-to-lymphocyte ratio (NLR), is a strong independent predictor of all-cause mortality in individuals with anemia."

**Lines 258-264: Contextualize NLR Threshold (1.475)**

Add to Discussion:

“While our identified NLR threshold of 1.475 is lower than thresholds reported in other populations (e.g., NLR >3.0 in critical illness (23), >5.0 in some cancers (24)), this likely reflects the unique vulnerability of anemic individuals to inflammation-driven morbidity. No prior study has validated this specific threshold in an anemia cohort, though similar non-linear relationships exist in CKD (NLR >2.2) and heart failure (NLR >3.8) (25). The lower threshold here suggests that anemia potentiates mortality risk at milder inflammatory levels, necessitating validation in external anemia cohorts. ”

**Line 278: Correct Study Design Terminology**

Original:

"First, while the NHANES dataset is comprehensive, its cross-sectional nature limits..."

Revised:

“First, while the NHANES dataset is comprehensive, its retrospective cohort design limits causal inference despite longitudinal mortality linkage.”

Gabriel Abbam (GA)
