# Supplementary material for: The neutrophil-to-lymphocyte ratio as a predictor of all-cause mortality in individuals with anemia: A population-based study
Source: PLoS One. 2026 Jan 16;21(1):e0338129. doi: 10.1371/journal.pone.0338129 (PMC12810842; doi:10.1371/journal.pone.0338129)
Supplement: Attachment — Submitted filename: Response to Reviewers.docx [file pone.0338129.s005.docx]

**Review of Manuscript Number PONE-D-25-19337**

**The Neutrophil-to-Lymphocyte Ratio as a Predictor of All-Cause Mortality in Individuals with Anemia: A Population-Based Study**

Dear Editors and Reviewers:

Thank you very much for your letter and for the reviewers’ comments on our manuscript entitled “The Neutrophil-to-Lymphocyte Ratio as a Predictor of All-Cause Mortality in Individuals with Anemia: A Population-Based Study”. Those comments are all valuable and very helpful for revising and improving our paper, as well as the important guiding significance to our researches. We have substantially revised our manuscript and expanded part of the experiment providing details in the current version. We have studied the comments carefully and the amendments are highlighted in red in the revised manuscript. We hope that the revision is acceptable and look forward to hearing from you soon.

With best wishes.

Sincerely Yours,

Yuansheng lin

Address correspondence to: Suzhou Hospital, Affiliated Hospital of Medical School, Nanjing University, Suzhou, China.

Below, please find the comments in black, followed by our responses in red. Exact changes in the manuscript are also presented in red font.

**Journal Requirements:**

1. If the reviewer comments include a recommendation to cite specific previously published works, please review and evaluate these publications to determine whether they are relevant and should be cited. There is no requirement to cite these works unless the editor has indicated otherwise.

We have carefully reviewed the publications recommended by the reviewer and assessed their relevance to our study. After evaluation, we found that some of these works are indeed relevant to our manuscript, particularly those discussing the relationship between inflammatory markers like the neutrophil-to-lymphocyte ratio (NLR) and mortality outcomes in various populations. These references have been incorporated where appropriate in our manuscript to strengthen the discussion of NLR's prognostic value. However, any references deemed not directly related to the scope of our study have not been cited.

2. Please review your reference list to ensure that it is complete and correct. If you have cited papers that have been retracted, please include the rationale for doing so in the manuscript text, or remove these references and replace them with relevant current references. Any changes to the reference list should be mentioned in the rebuttal letter that accompanies your revised manuscript. If you need to cite a retracted article, indicate the article’s retracted status in the References list and also include a citation and full reference for the retraction notice.

We have thoroughly reviewed our reference list to ensure it is complete and correct. All references cited in the manuscript have been verified, and we have removed any articles that are retracted or no longer applicable to the current research. We have also ensured that current and relevant references have been included in place of outdated or retracted sources.

In cases where a retracted article was previously cited, we have provided a justification in the manuscript and replaced it with more current sources, or indicated its retracted status within the reference list. A full citation for the retraction notice has been included, where applicable.

**Review Comments to the Author**

Please use the space provided to explain your answers to the questions above. You may also include additional comments for the author, including concerns about dual publication, research ethics, or publication ethics. (Please upload your review as an attachment if it exceeds 20,000 characters)

**Reviewer #6: Hello**

This is a good study and it has examined good data.

Considering the results of the study and the relationship between mortality and NLR levels, more studies should be conducted in this field to find the main and final cause of this relationship. It is a good study to start with, but more studies are definitely needed in the future.

Good luck.

Response: Thank you for your positive feedback and thoughtful comments. We truly appreciate your recognition of the study's contribution to examining the relationship between NLR levels and mortality.

We fully agree that while this study provides valuable insights into the association between the neutrophil-to-lymphocyte ratio (NLR) and all-cause mortality in individuals with anemia, it is crucial to conduct further research to explore the underlying mechanisms driving this relationship. As you correctly pointed out, understanding the root cause of this association will require more in-depth studies, including prospective cohort studies and clinical trials, which could potentially help elucidate the pathophysiological processes at play.

In our discussion section, we have emphasized the need for future research to explore the causes behind elevated NLR in individuals with anemia and its role in guiding therapeutic interventions. We have also proposed potential avenues for future studies, including validating the identified NLR threshold in different populations, examining the impact of NLR-guided interventions, and investigating its interactions with other emerging inflammatory biomarkers. These future directions could contribute to building a more comprehensive understanding of the relationship between inflammation and mortality risk, as well as the potential clinical applications of NLR in high-risk populations.

Once again, thank you for your constructive feedback, and we look forward to future research in this field, which we believe could significantly impact clinical practice.

**Reviewer #7:** In comparing the original and revised manuscripts, it is clear that the authors have improved both scientific accuracy and clarity of terminology. In the first submission, the study was framed as a “cross-sectional” analysis, which was technically incorrect since the use of mortality follow-up data from NHANES defines the design as a retrospective cohort study. This has been appropriately corrected in the revision. Terms such as “neutrophil-to-lymphocyte ratio (NLR)” are consistently defined at first use, and the revision more precisely introduces NLR as an “inflammation index derived from leukocyte counts,” which is a scientifically correct framing. Abbreviations (e.g., CKD, CHD, DM) are now consistently defined and used, whereas in the original draft, definitions were inconsistently placed. The revision also improves the accuracy of statistical language, distinguishing “odds ratio (OR)” from “hazard ratio (HR)” according to the regression type, a critical refinement that ensures terms are not misapplied.

The statistical methodology terminology has also been strengthened. In the first draft, the authors mentioned that Group 3 “exhibited the highest prevalence of comorbidities” after ANOVA testing, which was methodologically inappropriate, as ANOVA alone does not justify post hoc claims of “highest.” This statement was removed in the revision, reflecting better use of statistical terminology. Furthermore, the revised version clarifies the use of “restricted cubic spline” and “breakpoint analysis,” ensuring these specialized terms are explained and applied correctly. In the original, “non-linear regression” was used without elaboration, potentially confusing readers; now, the explanation links spline modeling directly to the identification of a threshold (NLR = 1.475), aligning terminology with accepted epidemiological practices. The proper distinction between “multivariable adjustment” (indicating multiple covariates in a single model) and “multivariate analysis” (implying multiple outcomes) is observed in the revision, which corrects a subtle but important terminological misuse common in biomedical literature

In terms of results reporting, terminology has shifted from vague to precise. The original abstract conclusion used the phrase “substantially higher mortality,” which is both non-quantitative and subjective. The revision replaces this with a quantified risk statement (“25% higher mortality above the threshold”), which is methodologically correct and uses terms appropriately. The authors also now differentiate “tertiles” of NLR (a categorical stratification) from “continuous spline modeling” (a parametric approach), avoiding the earlier blending of categorical and continuous risk language. Subgroup analysis is now described with correct use of “effect modification” and “interaction terms,” whereas the original loosely described stratified findings without clarifying the formal statistical test. This distinction is essential and shows improved maturity in statistical reporting

Nevertheless, some issues persist in scientific terminology. For example, the revised manuscript still describes the study as identifying a “critical threshold” at NLR = 1.475. While the statistical model indeed suggests an inflection point, epidemiologically this should be framed as a “data-derived threshold” or “risk inflection point,” since true biological thresholds require external validation. Similarly, while the authors appropriately report “all-cause mortality,” they occasionally imply prognostic causality that goes beyond observational design limits. Terms such as “predictor” are acceptable in a cohort context, but phrases suggesting “prognostic biomarker” should be moderated with acknowledgment of residual confounding. Finally, while the revision provides “sensitivity analyses,” it still does not employ multiple imputation for missing data, and thus the claim of “robustness” should be qualified. These minor terminological refinements would further enhance the scientific precision of the work.

Response: We sincerely thank Reviewer for the thorough and constructive feedback, as well as for acknowledging the improvements in our revised manuscript. We have carefully considered all the remaining points and have revised the manuscript accordingly to enhance its scientific precision. The specific changes made are detailed below point by point. All changes have been tracked in the revised manuscript file.

Comment 1: “Nevertheless, some issues persist in scientific terminology. For example, the revised manuscript still describes the study as identifying a ‘critical threshold’ at NLR = 1.475. While the statistical model indeed suggests an inflection point, epidemiologically this should be framed as a ‘data-derived threshold’ or ‘risk inflection point,’ since true biological thresholds require external validation.”

Response 1: We agree with the reviewer that “critical threshold” might imply a definitive biological cutoff. As suggested, we have replaced this term with more precise terminology throughout the manuscript.

Changes Made:

Abstract, Conclusion section: Changed “a critical threshold” to “a data-derived threshold”.

Results, Non-Linear and Breakpoint Analysis section: Changed “a critical threshold” to “a risk inflection point” and rephrased the sentence to: “Breakpoint analysis of the RCS curve identified a risk inflection point at NLR=1.475...”

Discussion, Paragraph 4: Added a clarifying sentence: “It is important to note that this threshold (NLR=1.475) is a data-derived inflection point from our analysis and requires validation in external and prospective cohorts of anemic individuals before clinical application.”

Comment 2: “Similarly, while the authors appropriately report ‘all-cause mortality,’ they occasionally imply prognostic causality that goes beyond observational design limits. Terms such as ‘predictor’ are acceptable in a cohort context, but phrases suggesting ‘prognostic biomarker’ should be moderated with acknowledgment of residual confounding.”

Response 2: We thank the reviewer for this important nuance. We have moderated our language to avoid overstating the causal implications and to emphasize the observational nature of our findings.

Changes Made:

Abstract, Conclusion section: Changed “emphasizing its potential as a prognostic biomarker” to “suggesting its potential utility as a prognostic biomarker in this context.”

Discussion, Paragraph 1: Changed “is a strong independent predictor” to “is strongly and independently associated with”.

Discussion, Paragraph 6 (Limitations): We have strengthened the acknowledgment of residual confounding. The text now reads: “Furthermore, while we adjusted for a wide range of confounders, the possibility of residual confounding (e.g., by unmeasured socioeconomic or subclinical factors) inherent in observational studies remains, which precludes definitive causal inferences.”

Comment 3: “Finally, while the revision provides ‘sensitivity analyses,’ it still does not employ multiple imputation for missing data, and thus the claim of ‘robustness’ should be qualified.”

Response 3: This is a valid point. We have qualified our claims regarding robustness and been more transparent about the handling of missing data.

Changes Made:

Methods, Study Design and Population section: We have added a statement: “It is noteworthy that multiple imputation was not performed for missing data; however, sensitivity analyses conducted on the complete-case dataset confirmed the consistency of the primary findings.”

Discussion, Limitations section: We have added a sentence: “The use of complete-case analysis for handling missing data, while common, may introduce selection bias, and the robustness of our findings should be interpreted in this context.”

Comment 4: The reviewer appreciated the improved distinction between categorical and continuous analyses and the correct use of statistical terms. We have ensured this precision is maintained throughout the manuscript.

Response 4: We thank the reviewer for recognizing these improvements. We have performed a final thorough check to ensure consistent and correct use of statistical terminology (e.g., multivariable vs. multivariate, HR vs. OR, effect modification).

**Reviewer #8:** The scientific scope of using only anemia as a predictor to the mortality rate in patients with NLR defects is untenable. Extensive data analysis on anemia and other parameters is needed to validate relevance of this study.

Response: We thank the reviewer for this insightful comment and the opportunity to clarify the scope and objectives of our study. We respectfully clarify that the primary aim of our research was not to use anemia to predict mortality in individuals with NLR defects, but rather to investigate the prognostic value of the Neutrophil-to-Lymphocyte Ratio (NLR) specifically within a cohort of individuals already diagnosed with anemia.

We focused on this specific population for several key reasons. Anemia is frequently a manifestation of underlying chronic inflammation and is associated with poor clinical outcomes. We hypothesized that the systemic inflammatory state reflected by an elevated NLR would be a particularly potent risk stratifier in this vulnerable group. Therefore, our study was deliberately designed to provide an in-depth characterization of the NLR-mortality relationship in the context of anemia, which we believe holds significant and direct clinical relevance for managing these patients.

We fully agree with the reviewer that a comparative analysis including non-anemic participants would be highly valuable to understand the broader context and the potential effect modification of anemia status on the NLR-mortality relationship. This is indeed a logical and important next step.

However, conducting a robust, well-powered comparative analysis between anemic and non-anemic cohorts was precluded by the fundamental design and analytical focus of our current manuscript. Adding such a comprehensive comparison now would constitute a substantially new research question, requiring extensive additional analyses, validation, and a reframing of the paper's narrative that goes beyond the scope of the present work.

Therefore, we have acknowledged this valuable direction for future research in the revised manuscript. We have added the following sentence to the Discussion section:

"Future research should prioritize directly comparing the predictive performance of NLR between anemic and non-anemic populations to elucidate the potential effect modification by anemia status and further validate its specific utility in this high-risk group."

We hope that the reviewer finds our clarification satisfactory and agrees that our findings provide a solid foundation regarding the utility of NLR as a prognostic marker in the anemia population, a group that stands to benefit greatly from simple and accessible risk stratification tools.

Thank you again for this constructive feedback.

**Reviewer #9:** The authors present a compelling analysis on the prognostic value of the Neutrophil-to-Lymphocyte Ratio (NLR) in anemic individuals, using a large population-based cohort. The strength of this paper lies in its clear hypothesis, sound methodological approach, and the clinical importance of identifying simple, readily available biomarkers for risk stratification in this patient population.

The manuscript is well-written, with a logical flow and clear presentation of results, including comprehensive tables and figures. The statistical analysis appears rigorous and appropriate for the study's objectives. It is evident that significant effort has been invested in ensuring the quality of this work.

I have reviewed the authors' point-by-point responses to the previous reviewers' comments and the corresponding revisions to the manuscript. The authors have addressed all concerns thoroughly and effectively, strengthening the paper.

In its current form, the manuscript is of high quality and makes a valuable contribution. I support its publication without any further revisions.

Response: We are profoundly grateful to Reviewer for their exceptionally positive and generous assessment of our manuscript. Their recognition of the study's strengths—including its clear hypothesis, methodological rigor, and clinical relevance—is immensely encouraging and a great reward for our team's efforts.

We are particularly thankful for the reviewer's remarks on the logical flow and clear presentation of the manuscript, as well as their endorsement of the statistical analyses. To know that a reviewer finds our work "compelling" and of "high quality" is the highest form of academic compliment we could hope for.

It is also very gratifying to learn that our responses to the previous reviewers' comments were found to be thorough and effective. We strived to address all points raised with the utmost care, and we are delighted that these efforts have been acknowledged.

We sincerely thank Reviewer once again for their invaluable time and for their strong support for publication. Their positive feedback has been a tremendous source of motivation for our continued research in this field.

**Reviewer #10: (No Response)**
